# Impact of iodine concentration and scan parameters on image quality, contrast enhancement and radiation dose in thoracic CT

**DOI:** 10.1186/s41747-020-00184-z

**Published:** 2020-09-11

**Authors:** Marian S. Solbak, Mette K. Henning, Andrew England, Anne C. Martinsen, Trond M. Aaløkken, Safora Johansen

**Affiliations:** 1 Faculty of Health Sciences, Oslo Metropolitan University, Pilestredet 48, 0130 Oslo, Norway; 2 Department of Radiology and Nuclear Medicine, Oslo University Hospital, Oslo, Norway; 3 School of Allied Health Professions, Keele University, Staffordshire, England; 4 Department of Diagnostic Physics, Oslo University Hospital, Oslo, Norway; 5 Faculty of Medicine, University of Oslo, Oslo, Norway; 6 Department of Cancer Treatment, Oslo University Hospital, Oslo, Norway

**Keywords:** Contrast media, Phantoms (imaging), Radiation dosage, Thorax, Tomography (x-ray computed)

## Abstract

**Background:**

We investigated the impact of varying contrast medium (CM) densities and x-ray tube potentials on contrast enhancement (CE), image quality and radiation dose in thoracic computed tomography (CT) using two different scanning techniques.

**Methods:**

Seven plastic tubes containing seven different CM densities ranging from of 0 to 600 HU were positioned inside a commercial chest phantom with padding, representing three different patient sizes. Helical scans of the phantom in single-source mode were obtained with varying tube potentials from 70 to 140 kVp. A constant volume CT dose index (CTDIvol) depending on phantom size and automatic dose modulation was tested. CE (HU) and image quality (contrast-to-noise ratio, CNR) were measured for all combinations of CM density and tube potential. A reference threshold of CE and kVp was defined as ≥ 200 HU and 120 kVp.

**Results:**

For the medium-sized phantom, with a specific CE of 100–600 HU, the diagnostic CE (200 HU) at 70 kVp was ~ 90% higher than at 120 kVp, for both scan techniques (*p* < 0.001). Changes in CM density/specific HU together with lower kVp resulted in significantly higher CE and CNR (*p* < 0.001). When changing only the kVp, no statistically significant differences were observed in CE or CNR (*p* ≥ 0.094), using both dose modulation and constant CTDIvol.

**Conclusions:**

For thoracic CT, diagnostic CE (≥ 200 HU) and maintained CNR were achieved by using lower CM density in combination with lower tube potential (< 120 kVp), independently of phantom size.

## Background

Computed tomography (CT) enables detailed evaluation of the vascular system by obtaining contrast-enhanced scans. In thoracic CT, numerous vascular conditions such as aneurysms, haemorrhage, dissection and malformations require the administration of iodinated contrast medium (CM) to improve differentiation between normal anatomy and pathology [1–3]. Even though contrast-enhanced CT has become an important diagnostic tool, challenges still exist regarding exposure to ionising radiation and the use of iodinated CM. Exposure to ionising radiation is known to be carcinogenic and associated with an additional risk of cancer [4–6]. Thus, CT scanning must always be considered in accordance with the ALARA principle (as low as reasonably achievable) as regarding the radiation dose [4]. Patients with impaired renal function are especially at risk of developing post-contrast acute kidney injury caused by iodinated CM. Since the impact of both CM volume and renal CM concentration is still under debate, it is essential to reduce the risks through CM optimisation [7–9].

The degree of contrast enhancement (CE) is an important factor when determining the diagnostic image quality of a CT examination. This is particularly important when evaluating small structures such as the coronary arteries [10] or evaluating the presence of metastatic disease in lung parenchyma [11]. Three different factors have been shown to influence CE: acquisition parameters, patient physiology, and CM-related factors [12, 13]. Most important is the iodine delivery rate (*i.e.,* the amount of iodine delivered per second) and the total iodine dose administered to the patient. For thoracic CT, high iodine concentrations (300 mg I/mL and above) have routinely been administered to achieve diagnostic levels of CE, but a higher iodine concentration itself does not result in a higher attenuation level when iodine delivery rate and total iodine dose are kept constant [3, 10, 14].

Multi-detector CT scanners enable large volume coverages in a short time. Using high pitch in combination with wider detector ranges and shorter rotation time allows image acquisition at peak arterial CE before venous circulation impacts resultant image quality. This allows for more effective identification of hyper-vascular tumours [15]. However, fast scanning restricts the CM volume and consequently the x-ray photon absorption. Alternatively, increasing iodine delivery rate may permit a reduction in CM volume and CM concentration, while maintaining diagnostic CE. A high iodine concentration is routinely used in CT angiography, to achieve opacification of around 250–300 HU in the thoracic aorta, and 300–350 HU in the coronary arteries [3, 10, 12, 13]. The target opacification required for routine chest CT is typically lower (150–200 HU for thoracic vessels), but often depends on preferences of the supervising radiologist [3, 16, 17].

Automatic tube voltage assistance techniques facilitate radiation dose reduction and improved visualisation of arteries. This is achieved by lowering the tube potential throughout the scan towards the k-shell energy level of iodine (33.2 keV), thereby increasing photoelectric effect [14, 18–20]. Scanning with a low tube potential and higher injection rate can allow a reduction of iodine concentration [13, 21] with the benefit of decreased contrast viscosity and a reduced risk of post-contrast acute kidney injury [10, 22]. This method has been reported to reduce the radiation and iodine dose by between 40–45% and 56–74%, respectively, without loss of image quality [23–27].

Many studies have investigated the feasibility of “double-low” techniques which combine low tube voltage with low CM densities/volume and/or CM concentration for aortic, coronary and pulmonary CT angiography. These studies have repeatedly proved “double-low” techniques to be beneficial by significantly reducing the iodine load and radiation dose [25, 28, 29]. However, to our knowledge, there is little known about the impact of CM densities/volume and varied tube potentials specifically for thoracic CT examinations. The aim of this study was to investigate the impact of variations in CM densities/volume for different tube potentials on contrast enhancement (CE), overall image quality and radiation dose in thoracic CT examinations.

## Methods

### Scan technique and phantom setup

**Fig. 1:**
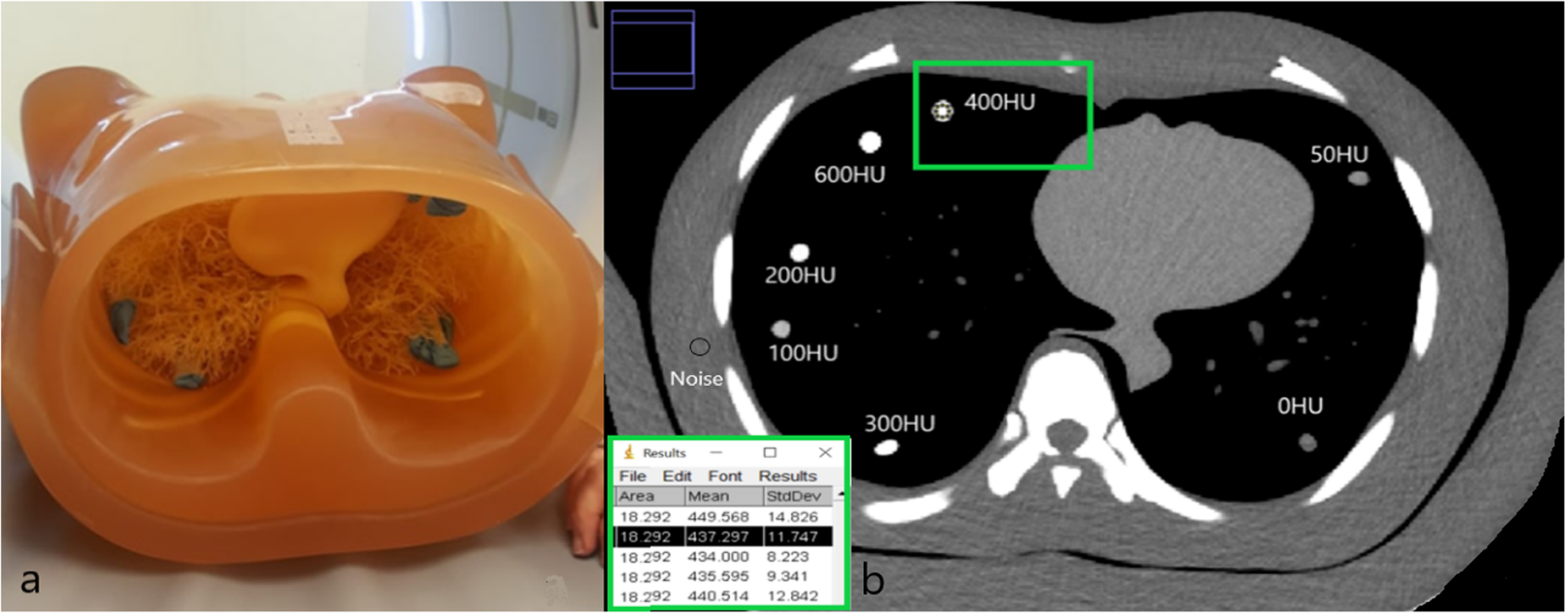
**a** Kyoto Kaguka Lungman Phantom (medium size) displaying the seven straws. **b** A single computed tomography (CT) image acquired using the phantom. This image includes the seven straws containing different iodine densities, resulting in specific HU values at 120 kVp when using a constant volume CT dose index

Seven drinking straws (0.8 cm in diameter, 24 cm in length), containing mixtures of saline solution and iodinated CM, were used to simulate blood vessels. Straws were placed in a circular pattern peripherally in the lung of a commercial anthropomorphic chest phantom (N1 Lungman, Kyoto Kagaku Co., Tokyo, Japan) (Fig. 1a). The phantom has been described by Afadzi and colleagues [30] and Gomi et al. [31] and has been also reported in other studies [32, 33]. Three phantom sizes were used. A set of anterior and posterior plates or “fat jackets” were added to simulate a large phantom (26 × 31 cm), the anterior plate was removed to simulate a medium phantom (23 × 31 cm), as illustrated in Fig. 1a, and no external plates were used for the small-sized phantom (20 × 27 cm).

A series of CT scans were performed using a General Electric Revolution CT scanner (GE Healthcare, Waukesha, WI, USA). Phantoms were scanned at 70, 80, 100, 120, and 140 kVp at 0.5 s/rotation. Due to restrictions in tube output when employing a short acquisition-time and low tube potentials, the rotation time was increased to 1 s/rotation when scanning the medium phantom at 70 kVp and scanning the large phantom at 70 and 80 kVp. A total of 30 CT acquisitions were performed with the scan parameters stated in Table 1. The first 15 acquisitions were acquired with automatic dose modulation. The following 15 acquisitions were scanned with a fixed mean volume CT dose index (CTDIvol) of 7 mGy (small phantom), 10 mGy (medium phantom), and 17 mGy (large phantom), maintaining a near constant level of tube output per rotation. For the large phantom, only 13 mGy was achieved for the 70 kVp tube potential due to tube current limitations.

**Table 1:** Details of the contrast medium specifics

Phantom size	Scan parameter tube voltage:	Constant CTDI					Dose modulation				
		70 kVp	80 kVp	100 kVp	120 kVp	140 kVp	70 kVp	80 kVp	100 kV	120 kV	140 kV
Large	Rotation time (s)	1	1	0.5	0.5	0.5	0.5	0.5	0.5	0.5	0.5
	mAs (range 80–500)	500	395	405	250	170	499	499	464	372	282
	CTDIvol (mGy)	13.17	16.6	16.68	16.6	16.43	6.59	10.11	14.79	16.6	17.59
Medium	Rotation time (s)	1	0.5	0.5	0.5	0.5	0.5	0.5	0.5	0.5	0.5
	mAs (range 80–500)	380	475	240	150	105	499	499	322	244	178
	CTDIvol (mGy)	10	9.98	9.89	9.96	10.15	6.09	8.14	8.98	10	11.6
Small	Rotation time (s)	0.5	0.5	0.5	0.5	0.5	0.5	0.5	0.5	0.5	0.5
	mAs (range 80–500)	500	330	170	105	70	484	630	219	106	123
	CTDIvol (mGy)	6.59	6.93	7.00	6.97	6.77	4.67	5.18	5.73	7.05	8.75

*CTDIvol* Volume computed tomography dose index. Scan technique: constant CTDIvol and dose modulation (SmartmA) using conventional, iterative reconstruction. Additional parameters: scan mode, helical; detector collimation, 80 × 0.625; pitch, 0.5; scan field of view, 50 cm; display field of view, 36 cm; reconstructed slice thickness, 2.5 mm; reconstruction kernel, standard; iterative reconstruction, adaptive statistical iterative reconstruction, ASIR-V 50%; noise index14.5 (scans with dose modulation)

### Assessment of contrast enhancement

Iodinated CM of 350 mg I/mL (Omnipaque, Iohexol, General Electric Healthcare, Oslo, Norway) was diluted in saline. When mixing the two components, equal amounts of saline were extracted, and CM was added to the prefilled 100 mL bottles of saline. At 120 kVp, 1.2 mL CM resulted in enhancement of 100 HU. Assuming a proportional relationship between CM and HU, the amount of CM necessary to obtain different CE levels was calculated (Table 2). For simplicity, in the “Results” and “Discussion” sections, iodine concentrations/iodine density will only be referred to as HU values (a specific HU) as established in Table 2.

**Table 2:** Details of the computed tomography scanning parameters

Iodine densities (mg I/mL → HU)	CM volume (350 mg I/mL)	Mixing ratio (CM: Saline solution)
0 → 0	0 mL	0:1
2.1 → 50	0.6 mL	1:165
4.2 → 100	1.2 mL	1:82
8.4 → 200	2.4 mL	1:41
12.6 → 300	3.6 mL	1:27
18.2 → 400	5.2 mL	1:18
26.6 → 600	7.6 mL	1:12

*CM* Contrast medium

### Image quality assessment

Image analyses were performed using the ImageJ software [34]. The ROIs were manually traced, and for each scan, a region of interest of 18.3 mm^2^ was placed in five slices (with 1 cm spacing) in each of the seven straws containing CM. The same approach was used to measure noise outside the lungs in the chest wall, in five slices (Fig. 1b). Measurements were repeated 35 times (5 × 7) for each scan and for the different tube potentials (70–140 kVp) and phantom sizes making a total of 600 circular regions of interest (Fig. 1). Image quality was assessed by calculating contrast-to-noise ratio (CNR) [35]. This study refers to enhancement of 200 HU and 120 kVp [3] as reference level. Images with CE ≥ 200 HU were considered diagnostically acceptable for thoracic CT scans [3, 13, 17, 36, 37].

### Dose assessment

The CTDIvol reported on the scanner after exposure was noted for each scan. The hospital CT quality assurance programme was carried out twice in 2018 and once in December 2019, by CT physicists. Measured CTDI in air was on all the three occasions within 10% from the vendor’s technical specification, and according to the institutions’ protocol for quality assurance, CTDIvol is then assumed to be within similar acceptance.

### Statistical analysis

Analysis was performed using SPSS Version 26 (IBM Inc, Armonk, NY, USA). Data was presented as mean ± standard deviation, minimum and maximum values. The non-parametric Kruskal-Wallis analysis of variance by ranks was also used to compare the difference in objective image quality. The *p* values < 0.05 were considered as statistically significant.

## Results

### CE assessment

**Fig. 2:**
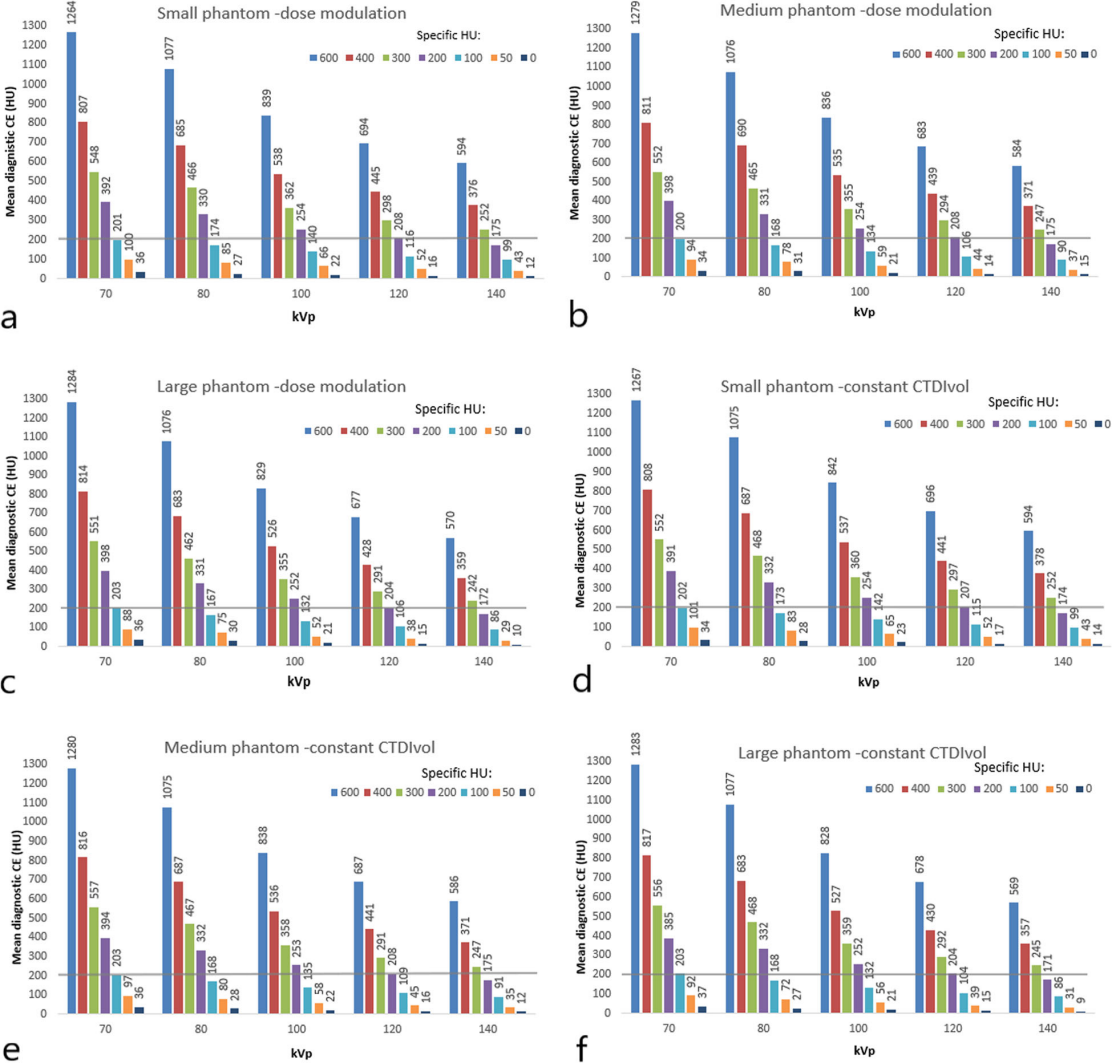
Measured contrast enhancement (CE) values as a result of employed specific HU values between 0 and 600 HU at 70–140 kVp when using dose modulation (**a**, **b** and **c**) and constant volume computed tomography dose index (CTDIvol) (**d**, **e** and **f**) for the three different phantom sizes. The horizontal line displays the reference diagnostic enhancement level of ≥ 200 HU

The mean diagnostic CE was higher for all CM densities and all three phantom sizes at lower tube potentials (70–100 kVp) when compared to the reference tube voltage of 120 kVp (Fig. 2). The diagnostic CE at 70 kVp and 80 kVp was 91% and 59% higher, respectively, when compared to acquisitions at 120 kVp. This was seen for both acquisitions using dose modulation, and for those with a constant CTDIvol for the reference CM density at 200 HU (Fig. 2). Our results show that using lower CM densities/specific HU at lower tube potentials (*e.g.,* 70 kVp) will result in higher diagnostic CE compared to acquisitions at 120 kVp (Fig. 2). These differences were statistically significant (*p* < 0.001). There was no significant difference in the measured CE between phantom sizes (*p* ≥ 0.494).

### Image quality assessment

**Fig. 3:**
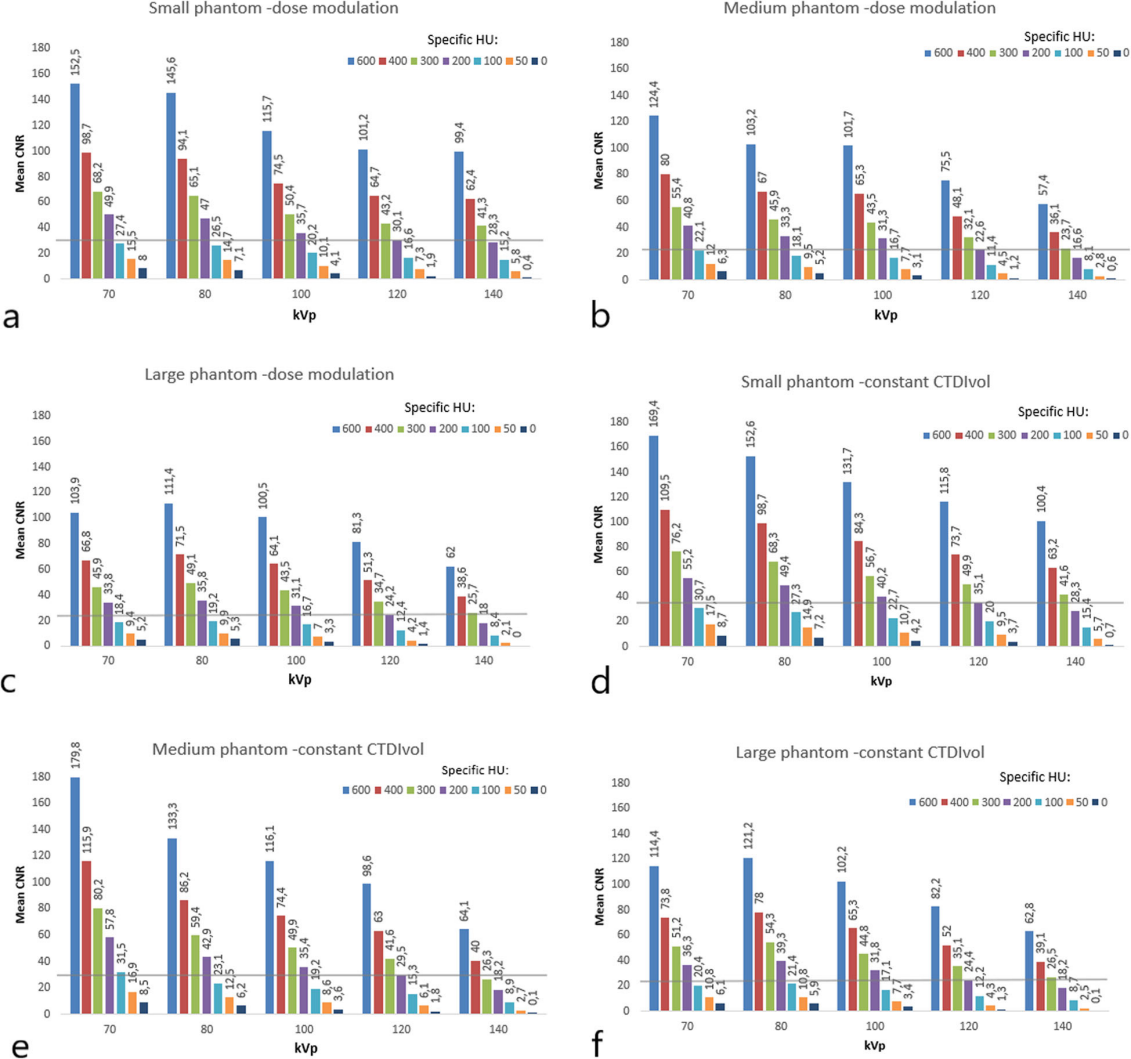
Mean contrast-to-noise values measured for the specific HU between 0 and 600 HU at tube potentials between 70–140 kVp when using dose modulation (**a**, **b** and **c**) and a constant volume computed tomography dose index (CTDIvol) (**d**, **e** and **f**) for the three different phantom sizes. The horizontal line displays the diagnostic contrast enhancement level ≥ 200 HU

For the medium-sized phantom, at the reference level (120 kVp, 200 HU), CNR started at 30 and 23 for the images obtained at constant CTDIvol and dose modulation, respectively, (Fig. 3). At the lowest tube voltage of 70 kVp, our results showed an increase in the CNR of 80% for the 200-HU CE using dose modulation, compared to the reference at 120 kVp. When using constant CTDIvol, our results showed that CNR was higher (96%) for 200 HU CE at 70 kVp *versus* 120 kVp. The higher CNR at lower tube voltages was seen for all measurements carried out in this study, independently of the scan technique and phantom size (Fig. 3).

Changes following lower CM density/specific HU, resulted in significantly higher CE and CNR (*p* < 0.001). However, when changing the tube potential, no significant differences (for the same CM density/specific HU) were observed in CE or CNR (*p* > 0.094). Again, this was seen for both dose modulation and for a constant CTDIvol (Figs. 2 and 3).

### Radiation dose

For scans with constant CTDIvol, the radiation dose was 7, 10, and 17 mGy for small, medium and large phantoms across the various tube potentials (Table 1). However, for the large phantom, the CTDIvol was 13 instead of 17 when scanning with 70 kVp due to technical limitations of the CT scanner. The radiation dose, when dose modulation was applied for the medium-sized phantom, showed a reduction of 10%, 19%, and 39% for 100 kVp, 80 kVp, and 70 kVp, respectively when compared to the reference tube voltage (120 kVp) (Table 1). For the large phantom, the radiation dose decreased with 11%, 39%, and 60% at 100 kVp, 80 kVp, and 70 kVp. Correspondingly, a dose reduction of 19%, 27%, and 34% was observed for the small phantom, across the respective tube potentials. Note that due to tube output limitations, the mAs was cut at a certain level for the large phantom at 70 and 80 kVp and the medium phantom at 70 kVp, resulting in lower doses than expected.

## Discussion

In this study, the impact of varying CM densities at different tube potentials on the overall image quality and diagnostic CE for thoracic CT was investigated. Our results show that increasing CM densities leads to significantly higher diagnostic CE and image quality (CNR). While a reduction in tube potential was shown to increase CE and CNR, these differences were not statistically significant, regardless of employed scan technique or patient size.

The clear benefits of low-kV protocols have been a favoured topic, with resulting radiation and CM dose savings between 40 and 60% reported, when reducing tube potential from 120 to 80 kVp [23–27]. Further dose savings and improved image quality have been reported, when combining low kV and optimised injection protocols which would reduce CM volume or concentration [14, 28, 36, 38]. The latter is demonstrated in the present phantom-based study. Reducing the tube voltage from 120 to 70 kVp for reference diagnostic CE of 200 HU resulted in increases in CNR of up to 80% and 96% using dose modulation and constant CTDIvol, respectively, in the medium phantom size. When lowering the tube potential, there is a debate regarding which approach is optimal for lowering the CM dose. Fleischmann et al. [14] have reported moderate iodine concentrations (300 mg I/mL) to be superior to higher CM concentrations (400 mg I/mL) for a constant iodine delivery rate when using 70 kVp to achieve sufficient CE over 300 HU [14]. The “double-low” approach has been favoured by several earlier studies [14, 28, 38–40] because of resultant lower effective doses and iodine doses with comparable image quality. According to the literature, a CE ≥ 200 HU in the thoracic region is clinically acceptable [3, 13]. For a routine chest CT, 60–70 mL of 350–370 mg I/mL CM has been suggested to be acceptable to achieve a CE of 150–200 HU [3].

The results of our study, despite investigating the impact of CM density on enhancement instead of CM concentration used in other studies, confirm the same tendency as reported in the study by Sun et al. [28]. They suggested greater potential for lowering the iodine load even in obese patients by 27%, scanning with 100 kVp instead of 120 kVp. Our investigation has reported a 50% reduction of CM density (medium phantom size) for the same diagnostic CE (200 HU) and CNR, by reducing tube potential from 120 to 70 kVp, using dose modulation as shown in Figs. 2 and 3. Our findings are also in agreement with the 51% CM density reduction reported by Thor et al. [27]. However, larger patient sizes as demonstrated in our study (see Fig. 3) may be a limitation with regards to the required tube output needed to achieve comparable CNR levels when using higher kVp values. Van Hamersvelt et al. [26] have shown a similar 40–60% iodine reduction, without loss of image quality using dual source and dual energy CT. In our study, a single source and single energy CT protocol was employed.

The CNR is primarily affected by CM signal and image noise, broadly becoming the most appropriate measure for investigating iodine-enhanced vessels and structures [27, 40]. When increasing contrast enhancement, by lowering photon energy towards the k-shell electron binding energy of iodine, more noise is accepted [41]. As shown in Fig. 3, the CNR increased from 22.6 to 40.8, approximately 80%, for the medium-sized phantom, when tube potential was reduced from 120 to 70 kVp (see Fig. 3). CNR (22.6 to 40.8) increased by 55.4% when using a fixed CTDIvol, inherently improving the image quality (see Fig. 3). However, these differences in CNR were not statistically significant, when using both scan techniques (*p* < 0.094). Patient size nevertheless has a great impact on image noise as photon penetration decreases in larger patients and a higher x-ray beam energy is required to achieve the same noise level [36, 39]. In our study, the image quality remained diagnostically acceptable independent of phantom size.

The CNR values resulting in a diagnostic CE ≥ 200 HU were above 23 and 30, with and without dose modulation, for the medium-sized phantom (Fig. 3). When compared to constant CTDIvol, dose modulation continuously reduces the tube current to patient/phantom attenuation profile, while maintaining a given noise index. This may cause an effective dose reduction of 53% according to Kok et al. [24] which supports our observation with only a slight change in CNR using dose modulation compared to constant CTDIvol (Fig. 3).

There are several limitations in our study. This was a phantom study, thus, no anatomical noise or artefacts caused by breathing and pulsation were present in the images. Furthermore, for the fixed parameter settings, the CTDIvol was lower for the 70 and 80 kVp levels for the large phantom due to technical limitations*.* Still, the systematic evaluation of different CM concentrations in the different phantom sizes for different scan techniques and dose levels would not be possible to obtain in a clinical setting due to patient radiation dose issues. Thus, performing a phantom study as the first step of systematic evaluation prior to a clinical study is needed to fully assess different scan techniques and available parameter settings. Each ROI placed inside the plastic straws and the chest wall was separated by air, influencing the calculation of objective image quality. No assessment of subjective image quality was conducted in this study. Therefore, to fully assess and validate the findings in this study, clinical studies including both objective and subjective image quality evaluation are needed to confirm our findings in routine clinical care. However, our results show that increased CE at lower tube voltages can be employed in clinical practice.

In conclusion, this study demonstrated that the combination of lower CM densities (specific HU) combined with lower tube potentials (*e.g.,* 70 kVp) resulted in improved CE enhancement (~ 90% higher), and maintained image quality (80% higher CNR) in chest CT when compared to acquisitions at 120 kVp. Using double-low method in thoracic CT examinations, CM density can be reduced by approximately 50% while maintaining CNR. Our findings were independent of scan technique and phantom size. To fully assess the potential of reduced CM densities for lower kVp in chest CT, clinical validation of the results from this study are needed.

## Data Availability

The datasets used and/or analysed during the current study are available from the corresponding author on reasonable request

## Abbreviations

CE: Contrast enhancement
CM: Contrast medium
CNR: Contrast-to-noise ratio
CT: Computed tomography
CTDIvol: Volume CT dose index
